# *Ganoderma lucidum *polysaccharides in human monocytic leukemia cells: from gene expression to network construction

**DOI:** 10.1186/1471-2164-8-411

**Published:** 2007-11-09

**Authors:** Kun-Chieh Cheng, Hsuan-Cheng Huang, Jenn-Han Chen, Jia-Wei Hsu, Hsu-Chieh Cheng, Chern-Han Ou, Wen-Bin Yang, Shui-Tein Chen, Chi-Huey Wong, Hsueh-Fen Juan

**Affiliations:** 1 Department of Life Science, National Taiwan University, Taipei 106, Taiwan; 2 Institute of Biotechnology, National Taipei University of Technology, Taipei 106, Taiwan; 3 Institute of Biomedical Informatics, National Yang-Ming University, Taipei 112, Taiwan; 4 School of Dentistry, National Defense Medical center, National Defense University, Taipei 114, Taiwan; 5 Institute of Molecular and Cellular Biology, National Taiwan University, Taipei 106, Taiwan; 6 Department of Electronic Engineering, National Taiwan University, Taipei, Taiwan; 7 Institute of Biological Chemistry and the Genomics Research Center, Academia Sinica, Taipei 115, Taiwan; 8 Institute of Biochemical Sciences, National Taiwan University, Taipei 106, Taiwan; 9 Department of Chemistry and The Skaggs Institute for Chemical Biology, The Scripps Research Institute, La Jolla, CA 92037, USA; 10 Institute of Biomedical Electronics and Bioinformatics, National Taiwan University, Taipei 106, Taiwan; 11 Center for Systems Biology and Bioinformatics, National Taiwan University, Taipei 106, Taiwan

## Abstract

**Background:**

*Ganoderma lucidum *has been widely used as a herbal medicine for promoting health and longevity in China and other Asian countries. Polysaccharide extracts from *Ganoderma lucidum *have been reported to exhibit immuno-modulating and anti-tumor activities. In previous studies, F3, the active component of the polysaccharide extract, was found to activate various cytokines such as IL-1, IL-6, IL-12, and TNF-*α*. This gave rise to our investigation on how F3 stimulates immuno-modulating or anti-tumor effects in human leukemia THP-1 cells.

**Results:**

Here, we integrated time-course DNA microarray analysis, quantitative PCR assays, and bioinformatics methods to study the F3-induced effects in THP-1 cells. Significantly disturbed pathways induced by F3 were identified with statistical analysis on microarray data. The apoptosis induction through the DR3 and DR4/5 death receptors was found to be one of the most significant pathways and play a key role in THP-1 cells after F3 treatment. Based on time-course gene expression measurements of the identified pathway, we reconstructed a plausible regulatory network of the involved genes using reverse-engineering computational approach.

**Conclusion:**

Our results showed that F3 may induce death receptor ligands to initiate signaling via receptor oligomerization, recruitment of specialized adaptor proteins and activation of caspase cascades.

## Background

*Ganoderma lucidum *(*G. lucidum*, Reishi or Ling-Zhi) has been used in traditional Chinese medicine as an anti-tumor medication or as an immuno-modulator. Many reports showed Reishi extracts to possess anti-proliferative effects on many cancers, such as acute myelogenous leukemia [1], lung cancer [2], breast cancer [3], colorectal cancer [4], bladder cancer [5] and prostate cancer [6,7]. A fucose-containing polysaccharide fraction (F3), isolated from the water-soluble Reishi extract, is able to stimulate spleen cell proliferation and cytokine expression [8-11]. Understanding how the molecular mechanism is responsible for the effects of F3 on cancer cells remains to be elucidated and will require whole-system approaches, since isolated single molecular studies have not, so far, been able to unlock cancer-system complexity. Microarray analysis is the first step in understanding integrated cell functions and cell-specific gene-expression profiles. The response of cells to external stimuli can be followed over a period of time by measuring the differences in global gene expression. Global transcription analysis provides a new approach to the description of complex biological phenomena [12-14]; it is also of great use in the field of cancer biology [15-19].

Networks of interacting proteins can provide researchers rudimentary understanding in cellular mechanisms; therefore, it is possible to understand the cellular functions of Reishi polysaccharide (F3) through their linkages to characterized receptors. In broader terms, networks of gene linkages offer a new view on the meaning of F3 function, and in time should provide us with a more in-depth understanding of the function of cells [20]. Traditionally, protein-polysaccharide interactions have been studied individually by genetic, biochemical or biophysical techniques. However, the speed of which new proteins are being discovered or predicted has created a need for high-throughput interaction-detection methods. Consequently, in the last two years, more efficient methods have been introduced to tackle the problem globally, and in turn provide researchers with vast amount of interaction data [21]. *In silico *(computational) interaction predictions derived from gene context analysis (gene fusion [22,23], gene neighborhood [24,25] and gene co-occurrences or phylogenetic profiles [26,27]) and chip-based analysis have been reported [28]. However, little knowledge has been obtained with regard to protein-polysaccharide interactions. Identifying protein-F3 interactions and constructing anti-cancer pathways are quite important in revealing the molecular mechanisms involved in anti-cancer activities.

Tumor necrosis factor-related apoptosis inducing ligand (TRAIL, also called Apo2L or TNFSF10) is capable of inducing apoptosis in cancer cells but not in normal cells [29]. It is possible that certain connection to the Apo2L signaling pathway contributes to anti-tumor activities. Apo2L seems to be a potential candidate for anti-cancer drug [30]. The four cellular receptors binding to Apo2L are death receptor 4 (DR4, also called TRAIL-R1), death receptor 5 (DR5, also called Apo2, TRAIL-R2, TRICK 2, TNFRSF10B or Killer), decoy receptor 1 (DcR1 or TRAIL-R3) and decoy receptor 2 (DcR2 or TRAIL-R4) [31-33]. Death receptors belonging to the tumor necrosis factor (TNF) receptor gene family are defined by cysteine-rich extracellular domains [34,35]. Signals induced by these ligand-receptor interactions serve the function of activating or inducing cell death by apoptosis.

In this study, we intend to find out whether F3 has similar interactions with death receptors that stimulate apoptosis pathways in leukemia cells. To study how leukemia cells are conditioned by F3, we carried out a dynamic analysis of gene expression in THP-1 cells, a monocytic leukemia cell line, with F3 treatment at different time points. In this article, we used oligonucleotide microarray and real-time quantitative PCR to detect dynamic gene expression profiles; and through bioinformatics approach, we also constructed a gene network. Finally, we illustrated possible molecular regulations of *Ganoderma lucidum *polysaccharides in human monocytic leukemia cells.

## Results and discussion

*G. lucidum *has been used for long time to modulate immune system and to prevent or treat various human diseases [36]. The biologically active compounds originally isolated and purified from *G. lucidum *were identified as polysaccharides, and the main fraction was designated as F3 [11]. Although the anti-tumor activity of *G. lucidum *associated with polysaccharides was well-established *in vitro *and *in vivo*, detailed mechanisms of how they work still await to be elucidated [36]. Understanding how polysaccharides (F3) stimulate anti-tumor effects in THP-1 cells is quite important. From gene expression to the construction of gene network, our results contribute to the understanding of the molecular mechanisms of F3 exertion on THP-1 cells. Two molecular mechanisms of F3-induced immunomodulation activities, including TLR4 and TLR2 signal pathways, have been studied [9,10]. In this study, we explored the gene expression and gene network induced by F3 in leukemia THP-1 cells, and aimed to reveal pathways critical in F3-induced anti-cancer activity.

### The induction of TNF-*α *in F3-treated human monocytic cells THP-1

**Figure 1:**
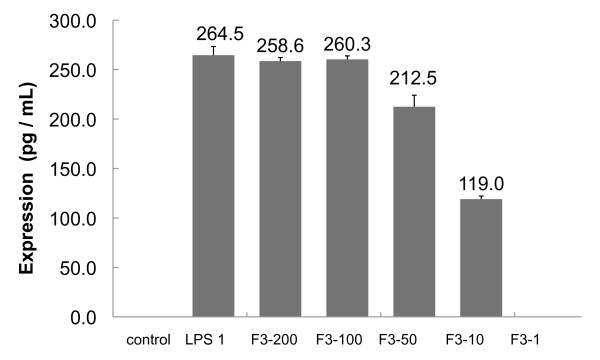
**The expression of TNF-*α *in F3- or LPS-induced THP-1 cells**. 10^5 ^cells/mL concentrations of THP-1 cells were seeded in 96-well microplates and incubated overnight. Then the cells (1.25 × 10^4^) were treated with F3 at dosages indicated as 1 *μ*g/mL, 10 *μ*g/mL, 50 *μ*g/mL, 100 *μ*g/mL, 200 *μ*g/mL, and with LPS at the dose of 1 *μ*g/mL, respectively. The same volume of medium was used as control After 24 hours, the supernatants were collected and in vitro TNF-*α *activity was determined using Human TNF-*α *Immunoassay Kit (Quantikine^®^, RD systems). TNF-*α *expression induced by F3 (100 *μ*g/mL and 200 *μ*g/mL) is similar to that of LPS (1 *μ*g/mL). From this TNF-*α *expression data, we calculated the EC_50 _(50% effect concentration) of F3-induced to be around 10 *μ*g/mL. The error bars indicate SD from triplicate independent experiments.

Upon the binding of TNF-*α *to TNFR1, monocytic cells are triggered to undergo apoptosis. This critical regulatory process is accomplished by activating the caspase cascade that results in the degradation of various important cellular proteins. Previous reports showed that lipopolysaccharide (LPS) could markedly stimulate the cytokine expression, especially TNF-*α *[37]. Compared with the TNF-*α *expression in LPS-induced THP-1 cells, we could estimate the optimal effect concentration of F3. We treated THP-1 cells with different F3 concentrations (1, 10, 50, 100, 200 *μ*g/mL) and LPS (1 *μ*g/mL) for 24 hours, and measured their TNF-*α *expressions. Figure 1 demonstrates that F3 was dose dependent in the activation of TNF-*α *expression. TNF-*α *expression stimulated by F3 at 100 *μ*g/mL and 200 *μ*g/mL was similar to that of LPS at 1 *μ*g/mL. From these TNF-*α *expression data, we calculated the EC_50 _(50% effect concentration) of F3-induced effectiveness to be around 10 *μ*g/mL. If 70% of TNF-*α *is expressed in F3-induced cells compared to LPS-induced cells, then 30 *μ*g/mL of F3 is required to achieve the same effectiveness. For further experiments, we used 30 *μ*g/mL of F3 to treat THP-1 cells.

### Apoptotic effect of F3 on THP-1 cells

**Figure 2:**
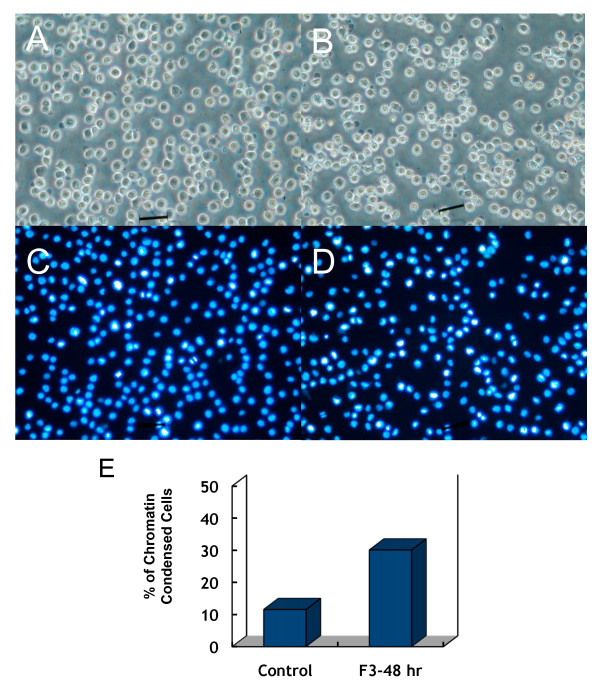
**Characterization of F3 induced cell death in human THP-1 cells**. Phase-contrast microscopy was used to detect the morphology of the control (A) and F3-treated THP-1 cells (B). Cell shrinkage, shape irregularity, and cellular detachment were observed in F3-treated cells, but not in the control. The control (C) and F3-treated THP-1 cells (D) were stained with 4, 6-diamidino- 2-phenylindole (DAPI). (E) The percentage of chromatin condensed cells. There was apparent difference in cell morphology between the un-treated and F3-treated THP-1 cells.

After THP-1 cells were treated with F3 (30 *μ*g/mL) for 48 hours, we observed the change of cell morphology under phase-contrast microscope. Differences in cell morphology can be detected between un-treated and F3-treated THP-1 cells. In Figure 2, representative photos of DAPI-staining results are shown. Cell shrinkage, one of cell death characteristics, happened in THP-1 cells after 48 hours treatment with F3 (Figure 2A and 2B). During cell apoptosis, an early event is the nuclear chromatin condensation, leading to the degradation of genomic DNA. DAPI nuclear staining was performed to check the apoptotic changes shown by cell morphology (Figure 2C and 2D). The percentage of chromatin condensed cells in F3-treated culture saw a significant increase (Figure 2E). Shrunken nucleus and apoptotic bodies in DAPI staining were features in determining whether cells had undergone apoptosis. These results indicated that incubation of THP-1 cells with F3 for 48 hours would lead to cell aggregations and apoptosis.

Recent studies showed *G. lucidum *to induce apoptosis in many cancer cells such as lung cancer cells [38], leukemia cells [39,40], murine skin carcinoma cells [41], colonic carcinoma cells [42], prostate cancer cells [6], and breast cancer cells [43]. In our study, we shared the same results with other reports, but more specifically in that F3 extracted from *G. lucidum *could in fact induce apoptosis of leukemia cells THP-1. In our effort to better understand the mechanism of this apoptotic effect, we used microarray, a high-throughput technique, to measure the dynamic gene expression in F3-treated THP-1 cells.

### Microarray analysis of overall gene expression in F3-induced THP-1 cells

**Figure 3:**
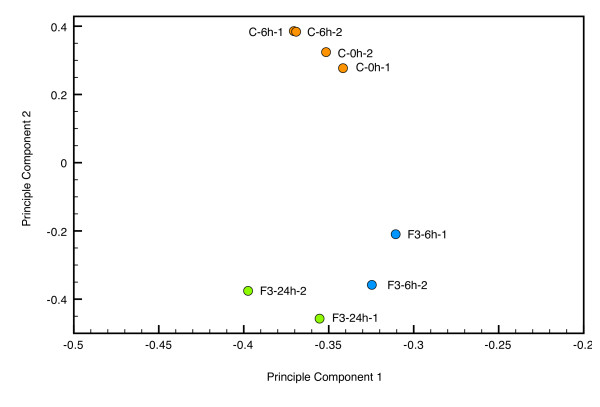
The scatter plot depicting the separation of control (0 and 6 hours) and F3-treated (6 and 24 hours) samples based on the first two principle components derived from the gene expression profiles measured by microarray experiments.

**Figure 4:**
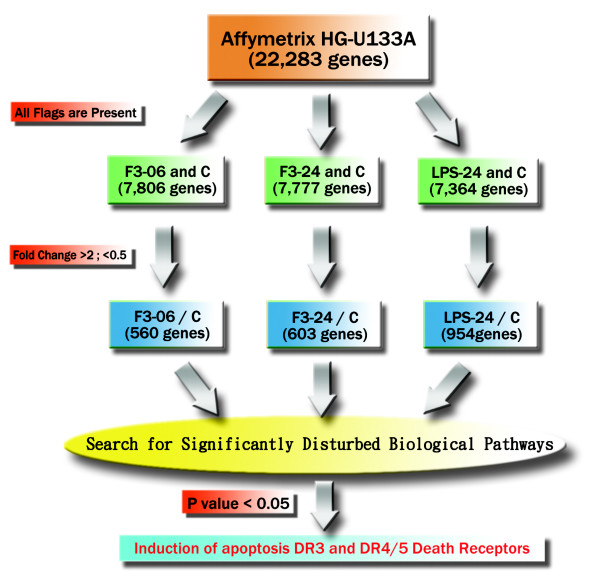
**The flow chart for the microarray data analysis**. We used Affymetrix HG-U133A chip GeneChip oligonucleotide microarray. Initial data analysis was performed using Affymetrix Microarray Suite v5.0 software, setting the scaling of all probe sets to a constant value of 500 for each GeneChip. Additional data analysis was performed using GeneSpring v 5.1 (Silicon Genetics Inc., Redwood City, California). Genes with a 2-fold change in differential expression between THP-1 control and F3- or LPS-treated THP-1 cells were selected for mapping significantly disturbed biological pathways. The pathway of apoptosis induction through the DR3 and DR4/5 death receptors was shown to be very significant in F3-treated THP-1 cells. F3–6 h and F3–24 h indicate the F3-treated THP1 cells after 6 hours and 24 hours, respectively. LPS-24 h indicates the LPS-treated THP1 cells after 24 hours. C-0 h and C-6 h indicate the control THP1 cells (without any treatment) in 0 hour and 6 hours, respectively.

**Figure 5:**
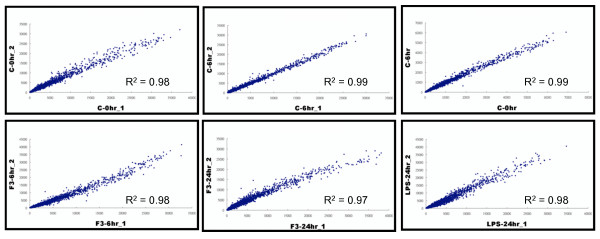
**Scatter plot of the gene expressions in the repeated microarray experiments**. The filtered probe intensities of gene expression of one experiment were plotted on the *x *axis while the intensities of the other experiment were plotted on the *y *axis. Each gene was represented by a single dot in the scatter plot. The upper three scatter plots showed no difference between control experiments for 0 hour and 6 hours. These results showed the consistency in duplicate microarray experiments.

In order to identify patterns of gene expression associated with apoptotic effect in THP-1 cells induced by F3, we performed a transcriptomic analysis on the THP-1 control and F3-treated or LPS-treated THP-1 cells by oligonucleotide microarray. In Figure 3, we observed clear separation of control (0 and 6 hours) and F3-treated (6 and 24 hours) samples after performing principle component analysis on the gene expression profiles measured by microarray experiments. Figure 4 shows the flow chart for our microarray data analysis. In Figure 5, the intensities of gene expression of one experiment were plotted on the *x *axis and the intensities of the other experiment on the *y *axis. A single dot represents one gene as shown in the scatter plots. The upper three scatter plots showed no difference between the control experiments for 0 hour and 6 hours. These results showed the consistency of our duplicate microarray experiments.

Genes with 2-fold change in gene expressions between THP-1 control and F3- or LPS-treated THP-1 cells were selected for mapping significantly disturbed biological pathways. The pathway of apoptosis induction through the DR3 and DR4/5 death receptors was observed to be significant (p < 0.05) in F3-treated THP-1 cells.

### Significant biological pathways related to F3-induced THP-1 cells

The differentially expressed genes were annotated to specific biological pathways. For each UniGene ID, we retrieved its biological pathways from either BioCarta [44] or KEGG [45] through the existing NCI CGAP gene information database [46]. Biological pathways were mapped and sorted on the order of matching significance using ArrayXPath [47]. Table 1 shows the significant biological pathways induced by F3 in THP-1 cells. In this study, four pathways, including TNFR2 signaling pathway, induction of apoptosis through DR3 and DR4/5 death receptors, NF-*κ*B signaling pathway, and toll-like receptor pathway may be involved in F3-induced cell death. The pathway of apoptosis induction through DR3 and DR4/5 death receptors was found to be the most significant pathway in F3-treated THP-1 cells for 6 hours. Detailed gene expression profiles of these four pathways after F3 or LPS treatments are shown in Table 2. Our findings implicated that multiple mechanisms may be involved in the anti-tumor effects of F3 extracted from *G. lucidum*.

**Table 1:** Significantly Disturbed Pathways of F3/LPS-treated THP-1 Cells

**Pathway**	**Identified**^a^	**p-value**	**q-value**
***F3 treatment after 6 hr***			
Induction of apoptosis through DR3 and DR4/5 Death Receptors	9/32 (37)	0	0.0044
Erythropoietin mediated neuroprotection through NF-kB	5/11 (17)	0.0002	0.0110

***F3 treatment after 24 hr***			
Inhibition of Matrix Metalloproteinases	3/9 (15)	0.0146	0.0265
Induction of apoptosis through DR3 and DR4/5 Death Receptors	6/32 (37)	0.0116	0.0265
IFN alpha signaling pathway	3/9 (25)	0.0146	0.0265
Chaperones modulate interferon Signaling Pathway	4/16 (34)	0.0140	0.0265
NF-kB Signaling Pathway	5/23 (49)	0.0112	0.0265
TNFR2 Signaling Pathway	5/18 (32)	0.0036	0.0265
CD40L Signaling Pathway	4/15 (27)	0.0110	0.0265
Bone Remodelling	4/14 (19)	0.0085	0.0265
B Lymphocyte Cell Surface Molecules	3/9 (10)	0.0146	0.0265
Double Stranded RNA Induced Gene Expression	3/10 (14)	0.0199	0.0321
Antisense Pathway	2/4 (13)	0.0208	0.0321
The information-processing pathway at the IFN-beta enhancer	4/15 (27)	0.0110	0.0440

***LPS treatment after 24 hr***			
B Lymphocyte Cell Surface Molecules	5/9 (10)	0.0006	0.0122
Erythropoietin mediated neuroprotection through NF-kB	5/11 (17)	0.0019	0.0191
Induction of apoptosis through DR3 and DR4/5 Death Receptors	8/32 (37)	0.0071	0.0356
IFN alpha signaling pathway	4/9 (25)	0.0063	0.0356
NFkB activation by Nontypeable Hemophilus influenzae	6/24 (43)	0.0194	0.0481
Toll-Like Receptor Pathway	7/34 (45)	0.0331	0.0481
TNFR2 Signaling Pathway	5/18 (32)	0.0207	0.0481
CD40L Signaling Pathway	4/15 (27)	0.0438	0.0481
IL-10 Anti-inflammatory Signaling Pathway	4/13 (17)	0.0266	0.0481
HIV-I Nef: negative effector of Fas and TNF	10/57 (77)	0.0338	0.0481
Neuropeptides VIP and PACAP inhibit the apoptosis of activated T cells	6/26 (43)	0.0283	0.0481
HIV-1 defeats host-mediated resistance by CEM15	2/3 (5)	0.0243	0.0481
Bone Remodelling	4/14 (19)	0.0346	0.0481
Neutrophil and Its Surface Molecules	3/8 (12)	0.0313	0.0481
Adhesion Molecules on Lymphocyte	3/9 (10)	0.0438	0.0481
GATA3 participate in activating the Th2 cytokine genes expression	5/16 (25)	0.0123	0.0481
Oxidative reactions of the pentose phosphate pathway	2/4 (4)	0.0457	0.0481
NF-kB Signaling Pathway	5/23 (49)	0.0558	0.0487
Chaperones modulate interferon Signaling Pathway	4/16 (34)	0.0543	0.0487
Double Stranded RNA Induced Gene Expression	3/10 (14)	0.0584	0.0487
Mechanism of Gene Regulation by Peroxisome Proliferators via PPARa(alpha)	9/54 (64)	0.0574	0.0487
FAS signaling pathway (CD95)	6/30 (34)	0.0536	0.0487
The information-processing pathway at the IFN-beta enhancer	4/15 (27)	0.0438	0.0487

^a ^The ratio between matching genes and total genes in this pathway

**Table 2:** Differentially expressed genes of F3- or LPS-induced THP-1 cells using oligonucleotide microarray

Pathway	Gene	Gene Description	Fold Change		
			F3–6 hr/C	F3–24 hr/C	LPS-24 hr/C
Induction of apoptosis through DR3 and DR4/5 Death Receptors	TNFSF10*	tumor necrosis factor (ligand) superfamily, member 10	9.9	10.1	18.4
	TNFRSF10B*	tumor necrosis factor receptor superfamily, member 10b	2.1	2.4	2.8
	CASP8*	caspase 8, apoptosis-related cysteine peptidase	1.3	1.3	1.6
	CASP10*	caspase 10, apoptosis-related cysteine peptidase	2.9	2.6	2.6
	BID	BH3 interacting domain death agonist	4.3	1.3	2.2
	BCL2*	B-cell CLL/lymphoma 2	0.6	0.7	0.7
	CYCS	cytochrome c, somatic	1.0	0.7	0.8
	APAF1	apoptotic peptidase activating factor	0.9	1.0	1.0
	CASP9	caspase 9, apoptosis-related cysteine peptidase	0.9	1.0	1.0
	CFLAR	CASP8 and FADD-like apoptosis regulator	3.1	2.3	4.2
	FADD*	Fas (TNFRSF6)-associated via death domain	0.8	1.0	0.9
	TRADD*	TNFRSF1A-associated via death domain	2.1	2.0	1.3
	TRAF2	TNF receptor-associated factor 2	1.1	0.9	0.6
	RIPK1*	receptor (TNFRSF)-interacting serine-threonine kinase 1	1.7	1.1	1.7
	MAP3K14	mitogen-activated protein kinase kinase kinase 14	1.6	1.1	1.9
	CHUK*	conserved helix-loop-helix ubiquitous kinase	0.9	0.8	1.0
	NFKBIA*	nuclear factor of kappa light polypeptide gene enhancer in B-cells inhibitor, alpha	12.6	6.6	10.5
	NFKB1*	nuclear factor of kappa light polypeptide gene enhancer in B-cells 1 (p105)	5.2	2.2	2.8
	BIRC2*	baculoviral IAP repeat-containing 2	1.5	1.0	1.7
	CASP3*	caspase 3, apoptosis-related cysteine peptidase	1.2	1.3	1.3
	CASP7*	caspase 7, apoptosis-related cysteine peptidase	3.4	2.1	2.7
	DFFB	DNA fragmentation factor, 40kDa, beta polypeptide (caspase-activated DNase)	0.4	1.0	0.5
	DFFA	DNA fragmentation factor, 45kDa, alpha polypeptide	0.8	1.1	0.9
	CASP6*	caspase 6, apoptosis-related cysteine peptidase	0.5	0.5	0.4
	LMNA	lamin A/C	1.6	1.6	1.6
	GAS2	growth arrest-specific 2	0.8	1.1	1.5
	SPTAN1	spectrin, alpha, non-erythrocytic 1 (alpha-fodrin)	1.3	1.3	0.9

NF-kB Signaling Pathway	TNFRSF1A	tumor necrosis factor receptor superfamily, member 1A	1.6	1.5	1.3
	FADD*	Fas (TNFRSF6)-associated via death domain	0.8	1.1	0.9
	TRADD*	TNFRSF1A-associated via death domain	2.1	2.0	1.3
	RIPK1*	receptor (TNFRSF)-interacting serine-threonine kinase 1	1.7	1.1	1.7
	TRAF6	TNF receptor-associated factor 6	1.3	1.1	1.1
	TLR4	toll-like receptor 4	1.3	1.2	0.9
	IRAK1	interleukin-1 receptor-associated kinase 1	0.6	0.8	0.6
	MYD88	myeloid differentiation primary response gene (88)	4.0	2.6	2.7
	IL1A	interleukin 1, alpha	3.2	1.2	2.4
	IL1B	interleukin 1, beta	63.2	17.5	53.1
	MAP3K7IP1	mitogen-activated protein kinase kinase kinase 7 interacting protein 1	0.7	1.0	0.8
	MAP3K7	mitogen-activated protein kinase kinase kinase 7	1.1	0.9	0.9
	CHUK*	conserved helix-loop-helix ubiquitous kinase	0.9	0.8	1.0
	MAP3K1	mitogen-activated protein kinase kinase kinase 1	0.8	1.2	1.1
	MAP3K14	mitogen-activated protein kinase kinase kinase 14	1.6	1.1	1.9
	IKBKB	inhibitor of kappa light polypeptide gene enhancer in B-cells, kinase beta	0.8	1.0	0.9
	IKBKG	inhibitor of kappa light polypeptide gene enhancer in B-cells, kinase gamma	1.2	1.2	1.2
	NFKBIA*	nuclear factor of kappa light polypeptide gene enhancer in B-cells inhibitor, alpha	12.6	6.6	10.5
	NFKB1*	nuclear factor of kappa light polypeptide gene enhancer in B-cells 1 (p105)	5.2	2.2	2.8
	RELA	v-rel reticuloendotheliosis viral oncogene homolog A, nuclear factor of kappa light polypeptide gene enhancer in B-cells 3, p65 (avian)	1.0	1.1	1.1

TNFR2 Signaling Pathway	LTA	lymphotoxin alpha	1.4	1.7	1.2
	TNFRSF1B	tumor necrosis factor receptor superfamily, member 1B	4.3	3.5	4.1
	RIPK1*	receptor (TNFRSF)-interacting serine-threonine kinase 1	1.7	1.1	1.7
	TRAF1	TNF receptor-associated factor 1	5.9	4.4	7.9
	TRAF2	TNF receptor-associated factor 2	1.1	0.9	0.6
	TRAF3	TNF receptor-associated factor 3	1.3	1.1	1.2
	TANK	TRAF family member-associated NFKB activator	2.3	1.9	2.9
	MAP3K14	mitogen-activated protein kinase kinase kinase 14	1.6	1.1	1.9
	MAP3K1	mitogen-activated protein kinase kinase kinase 1	0.8	1.1	1.1
	DUSP1	dual specificity phosphatase 1	2.7	1.5	4.0
	CHUK*	conserved helix-loop-helix ubiquitous kinase	0.9	0.8	1.0
	IKBKB	inhibitor of kappa light polypeptide gene enhancer in B-cells, kinase beta	0.8	0.9	0.9
	IKBKG	inhibitor of kappa light polypeptide gene enhancer in B-cells, kinase gamma	1.2	1.2	1.2
	IKBKAP	inhibitor of kappa light polypeptide gene enhancer in B-cells, kinase complex-associated protein	0.9	1.0	0.7
	TNFAIP3	tumor necrosis factor, alpha-induced protein 3	12.7	5.4	11.4
	NFKBIA*	nuclear factor of kappa light polypeptide gene enhancer in B-cells inhibitor, alpha	12.2	6.6	10.5
	NFKB1*	nuclear factor of kappa light polypeptide gene enhancer in B-cells 1 (p105)	5.2	2.2	2.8

Toll-like Receptor Pathway	TLR2	toll-like receptor 2	1.2	2.1	2.6
	CD14	CD14 antigen ; CD14 antigen	0.5	15.2	16.9
	MYD88	myeloid differentiation primary response gene (88)	4.0	2.6	2.7
	TLR4	toll-like receptor 4	1.3	1.2	0.9
	LY96	lymphocyte antigen 96	0.6	5.3	6.3
	TOLLIP	toll interacting protein	1.0	1.1	1.2
	IRAK1	interleukin-1 receptor-associated kinase 1	0.6	0.8	0.6
	TRAF6	TNF receptor-associated factor 6	1.3	1.1	1.1
	EIF2AK2	eukaryotic translation initiation factor 2-alpha kinase 2	5.1	4.0	4.5
	SITPEC	signaling intermediate in Toll pathway, evolutionarily conserved	0.8	0.7	0.4
	MAP3K1	mitogen-activated protein kinase kinase kinase 1	0.8	1.2	1.1
	CHUK*	conserved helix-loop-helix ubiquitous kinase	0.9	0.8	1.0
	IKBKB	inhibitor of kappa light polypeptide gene enhancer in B-cells, kinase beta	0.8	1.0	0.9
	IKBKG	inhibitor of kappa light polypeptide gene enhancer in B-cells, kinase gamma	1.2	1.2	1.2
	NFKBIA*	nuclear factor of kappa light polypeptide gene enhancer in B-cells inhibitor, alpha	12.6	6.6	10.5
	MAP3K7	mitogen-activated protein kinase kinase kinase 7	1.1	0.9	1.0
	MAP3K7IP2	mitogen-activated protein kinase kinase kinase 7 interacting protein 2	1.6	1.2	1.4
	MAP3K7IP1	mitogen-activated protein kinase kinase kinase 7 interacting protein 1	0.7	1.0	0.8
	MAP2K3	mitogen-activated protein kinase kinase 3	2.2	1.5	1.9
	MAP2K6	mitogen-activated protein kinase kinase 6	0.7	1.1	0.8
	MAPK14	mitogen-activated protein kinase 14	0.6	0.9	0.8
	MAP2K4	mitogen-activated protein kinase kinase 4	0.9	1.0	1.0
	MAPK8	mitogen-activated protein kinase 8	2.6	1.2	1.5
	JUN	v-jun sarcoma virus 17 oncogene homolog (avian)	7.9	1.3	2.6
	FOS	v-fos FBJ murine osteosarcoma viral oncogene homolog	1.5	2.3	1.8
	ELK1	ELK1, member of ETS oncogene family	0.9	1.4	1.5

*These genes have been validated by Q-PCR.

**Figure 6:**
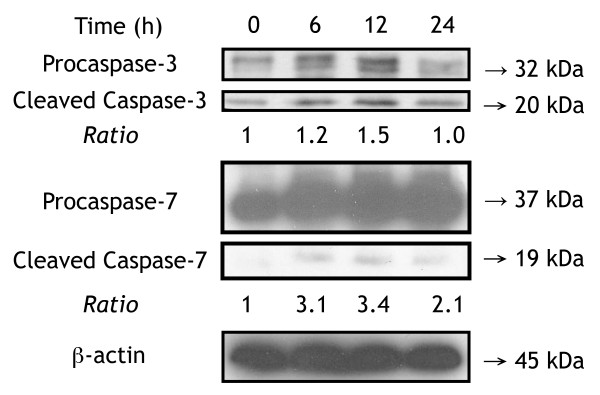
CASP3 and CASP 7 were cleaved into active forms after F3 treatment in THP-1 cells. After THP-1 cells were treated with F3 for 0, 6, 12, 24 hours, we detected proforms and active forms of CASP3 and CASP7 using western blotting. CASP3 and CASP7 were activated after F3 treatment.

In the pathway of apoptosis induction through DR3 and DR4/5 death receptors, 9 genes were found to be up-regulated and 2 to be down-regulated among the 27 genes in F3-treated THP-1 cells after F3 treatment for 6 hours. The 9 up-regulated genes include tumor necrosis factor (ligand) superfamily, member 10 (TNFSF10 or TRAIL), tumor necrosis factor receptor superfamily, member 10b (TNFRSF10B or DR5), caspase 10, apoptosis-related cysteine peptidase (CASP10), BH3 interacting domain death agonist (BID), CASP8 and FADD-like apoptosis regulator (CFLAR), TNFRSF1A-associated via death domain (TRADD), nuclear factor of kappa light polypeptide gene enhancer in B-cells inhibitor, alpha (NFKBIA), nuclear factor of kappa light polypeptide gene enhancer in B-cells 1 (NFKB1), and caspase 7, apoptosis-related cysteine peptidase (CASP7); all of these genes are involved in cell death. Two down-regulated genes were DNA fragmentation factor, 40kDa, beta polypeptide (caspase-activated DNase, DFFB) and caspase 6, apoptosis-related cysteine peptidase (CASP6). CASP6 cleavage by caspase-3 (CASP3), caspase-8 (CASP8) or -10 (CASP10) generates the two active subunits. In the microarray gene expression results, CASP3 and CASP8 showed no significant difference after F3 treatment. However, in our Q-PCR gene expression results, CASP8 showed a significant up-regulated expression. CASP3 and CASP7 were cleaved into their active forms after F3 treatment as shown in Figure 6. In summary, F3 may bind to death receptor 4/5, and activate downstream apoptosis-related cysteine peptidase such as CASP8, CASP3 and CASP7, leading to the apoptosis of THP-1 cells.

The NF-*κ*B signaling pathway includes 21 genes among which 6 genes were up-regulated after treatment with F3. These six genes were TRADD, myeloid differentiation primary response gene (88) (MYD88), interleukin 1, alpha (IL1A), interleukin 1, beta (IL1B), NFKBIA and NFKB1. All of these genes are involved and are crucial in cell death. IL1B at low concentration induced strong apoptotic responses as revealed by caspase-8 activation and DNA fragmentation [48]. MYD88 is an adapter protein involved in Toll-like receptor and IL-1 receptor signaling pathways in the innate immune response and acts via interleukin-1 receptor-associated kinase (IRAK1) and TNF receptor-associated factor 6 (TRAF6), leading to NF-*κ*B activation, cytokine secretion and inflammatory responses. MYD88 can also induce IL-8 transcription and may be involved in myeloid differentiation. TRADD was observed to be overexpressed over 25-fold after F3 treatment for 3 hours. TRADD is an adapter protein in the tumor necrosis factor receptor superfamily, in which member 1A (TNFRSF1A/TNFR1) specifically associates with the cytoplasmic domain of activated TNFRSF1A/TNFR1, mediating its interaction with Fas (TNFRSF6)-associated via the death domain (FADD) [31]. Overexpression of TRADD leads to two major TNF-induced responses, apoptosis and the activation of NF-*κ*B. NF-*κ*B is a transcription factor regulating the expressions of a large number of genes critical in apoptosis regulation. NF-*κ*B is strongly activated shortly after TNF engagement with TNFR1, generating a pro-survival signal that must be overcome in many cell types for TNF to induce apoptosis [31]. Many reports also showed that Apo2L/TRAIL could activate NF-*κ*B [29,49]. In our results, activation of the NF-*κ*B signaling pathway caused further THP-1 cell death after F3 treatment.

The TNFR2 signaling pathway includes 17 genes among which 7 genes were differentially expressed in F3- or LPS-treated THP-1 cells. All of these seven genes were up-regulated after treatments with F3 or LPS, as shown in Table 2. TNF binds to two different receptors, TNF receptor 1 (TNFR1) and TNFR2 (TNFRSF1B). TNFR2 is produced by activating lymphocytes and can be cytotoxic to many types of tumors and cells. TNFR2 expression is to mediate the anti-tumor effect of TNF, and NO is necessary for this process, possibly by inhibiting angiogenesis in the tumor [50]. Like TNFR2, TNF receptor-associated factor 1 (TRAF1) was also up-regulated in F3- or LPS-treated THP-1 cells. TRAF1 is an adapter protein and signal transducer that links members of the TNFR family to different signaling pathways by association with the receptor cytoplasmic domain and kinases. TRAF1 is involved in apoptosis by mediating the activation of NF-*κ*B and c-Jun N-terminal kinase (JNK) [51]. TRAF family member-associated NF-*κ*B activator (TANK) acts as a regulator of TRAF function by maintaining TRAF in a latent state. Alpha-induced protein 3 (TNFAIP3), a tumor necrosis factor, was induced by TNF-*α *and identified as a regulatory component of a putative cytoplasmic signaling cascade that mediates NF-*κ*B activation in response to DNA damage [52].

Toll-like receptor pathway was observed to be significant (p < 0.05 and q < 0.05) in LPS-treated but not in F3-treated THP-1 cells (Table 1). This pathway includes 27 genes among which six and seven genes were differentially expressed in F3- and LPS-treated THP-1 cells, respectively. After F3 treatment for six hours, MYD88, eukaryotic translation initiation factor 2-alpha kinase 2 (EIF2AK2), NFKBLA, mitogen-activated protein kinase kinase 3 (MAP2K3), mitogen-activated protein kinase 8 (MAPK8) and v-Jun sarcoma virus 17 oncogene homolog (avian) (JUN) were up-regulated and CD14 antigen (CD14) was down-regulated. Interestingly, CD14 was up-regulated after treatments with LPS and F3 for 24 hours. CD14 cooperates with TLR4 to mediate the innate immune response to LPS and acts via MYD88 and TRAF6, leading to NF-*κ*B activation, cytokine secretion and the inflammatory response. Lymphocyte antigen 96 (LY96) was up-regulated after treatment with F3 or LPS for 24 hours. The downstream signaling pathway used by toll-like receptors are similar to that used by IL-1 receptors, which is activating the IL-1 receptor associated kinase (IRAK) through the MYD88 adaptor protein, and signaling through TRAF-6 and protein kinase cascades to activate NF-*κ*B and Jun [53]. In conclusion, the expression of these genes in the toll-like receptor pathway may implicate their associations with THP-1 apoptosis after F3 or LPS treatment.

### Comparisons between oligonucelotide microarray and Q-PCR results

**Figure 7:**
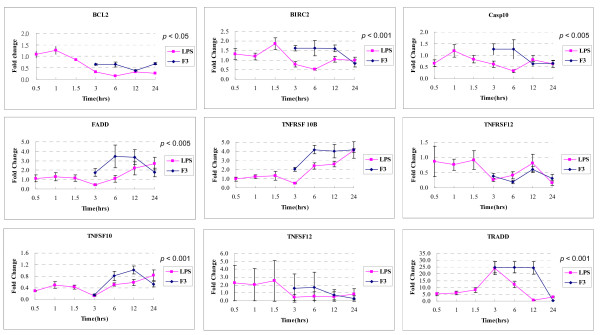
Fold change in time-course gene expression of F3- or LPS-induced THP-1 cells by Q-PCR. First, THP-1 cells were treated with F3 or LPS for different periods of time and were collected at different time points. Total RNA was isolated from cell lines using TRIzol reagent. First-strand cDNA synthesis were performed with 5 *μ*g of total RNA in a volume of 20 *μ*l with 1 *μ*l ThermoScript™ Reverse Transcriptase (Invitrogen) and 1 *μ*l oligo(dT). Extracted first-strand cDNAs were analyzed using BioRad iCycler iQ Real-Time Detection System with SYBR Green dye (Molecular Probes, Eugene, OR). Software designed for the BioRad iCycler will aid in analyzing collected data. mRNA expression of these genes were normalized to RNA content for each sample by using GADPH gene products as internal controls. Relative expression was calculated as the ratio of expression from each F3-treated THP-1 cells in comparison to untreated THP-1 cells (control). The error bar came from n > 3. The *p *values indicate the statistical significance of different time-course gene expression profiles between F3 and LPS treatment.

**Figure 8:**
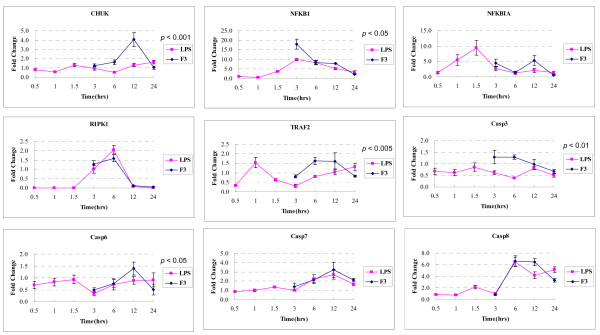
Fold change in time-course gene expression of F3- or LPS-induced THP-1 cells by Q-PCR. (continued from Figure 7)

**Figure 9:**
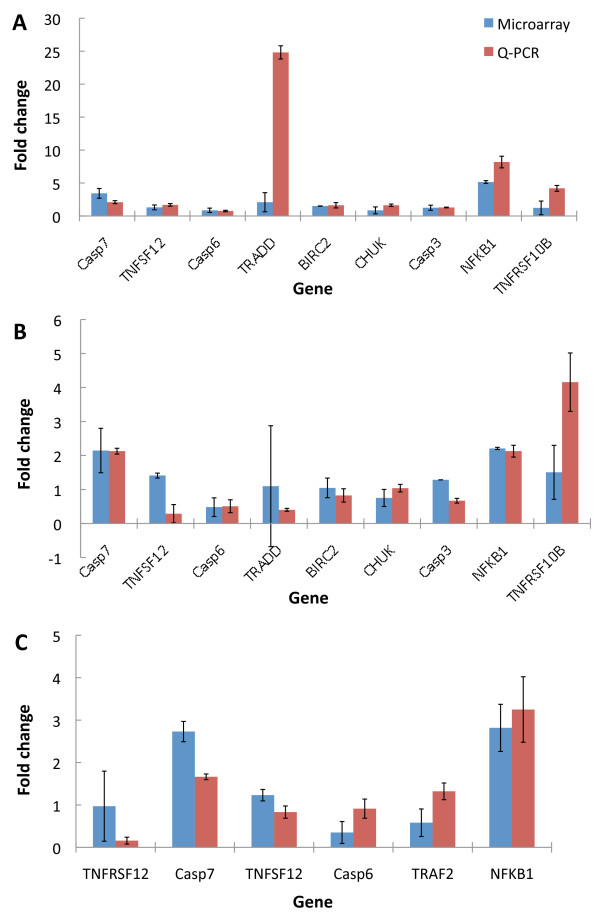
**Comparisons between oligonucelotide microarray and Q-PCR results**. (A) THP-1 cells were treated with F3 for 6 hours (A) and 24 hours (B) and with LPS for 24 hours (C). The gene expressions showed consistent trends between the microarray and Q-PCR results. The error bar came from n > 3.

To further validate our findings from the microarray analysis, we selected a set of genes known for their involvement in apoptosis through death receptors, and carried out gene expression studies using Q-PCR. 18 gene expressions related to the death receptor pathway were examined between F3- or LPS-treated THP-1 cells for 3, 6, 12, and 24 hours. mRNAs were reverse-transcribed and amplified through Q-PCR using primers specific for each gene of interest; the housekeeping gene, GAPDH, was used as internal control. Each experiment was repeated three times. The results are shown in Figure 7 and 8. Significance analysis of Q-PCR measurements was performed by EDGE software package [54]. There were nine significantly differential gene expressions in F3-treated THP-1 cells, including TNFRSF10B, CASP7, CASP6, TRADD, CASP3, TNFSF12, baculoviral IAP repeat-containing 2 (BIRC2/c-IAP), conserved helix-loop-helix ubiquitous kinase (CHUK/IKK*α*), and NF-*κ*B (NFKB1); whereas in LPS-treated THP-1 cells these were TNFRSF12, CASP7, TNFSF12, CASP6, TRAF2, and NFKB1. Figure 9 shows that nearly all genes are consistent between the microarray and Q-PCR data.

We compared the 18 gene expressions in the time courses after F3 or LPS treatments, as shown in Figure 7 and 8. Statistically significant difference of time-course gene expression profiles between F3 and LPS treatment was observed for TRADD, CHUK, TRAF2, BIRC2, TNFSF10, FADD, CASP3, CASP10, CASP6, BCL2, and NFKB1, while other genes exhibited similar expression trends. These results suggest that THP-1 cells have similar but different gene expression response to F3 and LPS treatments.

### Construction of cell death gene networks and possible pathways involved in F3-induced THP-1 cell death

**Figure 10:**
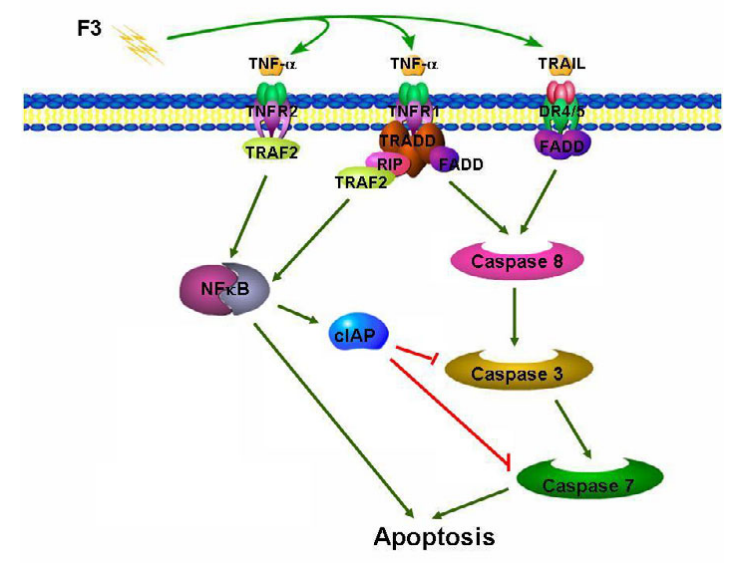
**Proposed F3-induced cell death pathways in THP-1 cells**. Based on our self-developed software for the reconstruction of gene networks in addition to literature research, we proposed plausible cell death pathways induced by F3 in THP-1 cells. F3 may induce death receptor ligands (TNF-*α *and TRAIL) to initiate signaling via receptor oligomerization, recruitment of specialized adaptor proteins and activation of caspase cascades. Lastly, cell shrinkage and apoptosis occur.

Based on our time-course Q-PCR data, we constructed the cell death gene network in F3-treated THP-1 cells by our self-developed software tool, BSIP [55]. Assuming that gene regulatory network follows the S-system mathematical model [56], we used BSIP to estimate the modeling parameters, identify the optimized structure, compare the concordance, and infer a plausible regulatory network. Moreover, we established the interrelated apoptosis pathways initiated by F3 in THP-1 cells using our concurrent findings, as illustrated in Figure 10. Death receptors, which belong to tumor necrosis factor (TNF) gene superfamily, are cell surface receptors that transmit apoptosis signals and play a crucial role in apoptosis and cell survival. Our characterized death receptors are TNFR1 (tumor necrosis factor receptor-1), TNFR2 (tumor necrosis factor receptor-2), and DR4/5 (also called TRAIL-R1/2) [31]. TNF-*α *and TNFSF10, also called TRAIL, exhibit an increase in gene expression in F3-treated THP-1 cells. When TNF-*α *binds to TNFR1, adaptor protein TRADD recruitment follows, and then it interacts with another death domain-containing molecule FADD, leading to the subsequent cleavage of pro-caspase-8 [57]. The binding of TRAIL to DR4/5 also induces the recruitment of FADD and pro-caspase-8 of auto-proteolytic activation [37]. Interactions between pro-caspase-8 and FADD result in further activation of caspase-3 and caspase-7, and also the initiation of apoptosis [58]. In addition to apoptotic signals, recruitment of TRAF2 through TNFR2 binding TNF-*α *activates NF-*κ*B, thus producing anti-apoptotic signals [59]. TNFR1 also activates anti-apoptotic NF-*κ*B. NF-*κ*B-dependent activation of *TRAF2*, *RIPK1*, and *BIRC2 *gene expressions seemed to support this because TRAF2 and RIP interacting with TRADD prevented caspase-8 activation, while c-IAPs inhibited caspase-3 and caspase-7 activation [60]. When NF-*κ*B is activated, I*κ*B is phosphorylated by protein kinase IKK and this phosphorylation serves as a signal for the ubiquitination and degradation of I*κ*B. Free NF-*κ*B dimers are released and translocated to the nucleus, where they enhance the transcription of target genes. NF-*κ*B activation and I*κ*B degradation prompt cell survival signals and mediate immune responses [61]. In contract, NF-*κ*B is a transcription factor that regulates expression of a large number of genes critical for the regulation of apoptosis, and many reports have showed that Apo2L/TRAIL can activate NF-*κ*B [29,49]. All in all, NF-*κ*B activation can promote apoptosis or survival, depending on the cellular contents [49].

## Conclusion

In conclusion, we performed time-course microarray analysis and Q-PCR assays for measuring gene expression profiles of F3-treated THP-1 cells. Integrating the expression data, we applied computational modeling to infer plausible gene regulatory networks involved in F3-induced cell death. Our results suggested that F3 might mimic/induce death receptor ligands such as TNF-*α *and TRAIL to initiate signaling via death receptor oligomerization, recruitment of specialized adaptor proteins, and the activation of caspase cascade, followed by cell shrinkage and apoptosis. This study confirmed microarray analysis to be a powerful tool for demonstrating gene expressions related to the efficacy of anti-tumor drugs such as *Ganoderma lucidum *polysaccharides, F3, on tumor growth. This approach opens a different way of elucidating the molecular mechanisms for anti-tumor compounds or drugs in cancer cells. Information in this study may present a powerful tool for cancer diagnosis and therapy.

## Methods

### Materials

Crude Reishi extract (prepared via alkaline extraction (0.1N NaOH), neutralization and ethanol precipitation) was obtained from Pharmanex Co. (CA, USA). All chemicals and reagents were from Sigma-Aldrich Co., unless indicated.

### Purification of Reishi extract

Crude Reishi powder (obtained from Pharmanex Co.) 6 g was dissolved in 120 mL of dd water, stirred at 4°C for 1 hour, and centrifuged (1000 rpm) for 1 hour to remove the insoluble. The resulting solution was concentrated at 40~ 50°C to give a small volume which then was lyophilized to generate 5 g (83%) powder of dark-brown color. This water soluble residue was stored at -20°C for further purification.

### Isolation of the F3 fraction of Reishi polysaccharide [8]

F3 was isolated from the dark powder of water soluble residue of Reishi polysaccharide. The procedure during chromatography was maintained at 4°C in cool room. The 2.1 g sample was dissolved in a small volume of Tris buffer (pH7.0, 0.1N) containing 0.1% sodium azide, and purified by gel filtration chromatography using a Sephacryl S-500 column (95 × 2.6 cm) with 0.1N Tris buffer (pH 7.0) as the eluent. The flow rate was set at 0.6 mL/min, and 6.0 mL per tube was collected. After the chromatography, each fraction was subjected to phenol-H_2_SO_4 _method to detect the content of sugar in each tube. Five fractions were collected (fractions 1–5), F3 fraction was concentrated at 40~ 50°C to give a small volume in rotary vapor, which was dialyzed or purified through G-50 column to remove excessive salt and sodium azide and was then lyophilized to give 520 mg (25%) of F3.

### THP-1 cell culture and induction of cell death

The monocyctic cell-line THP-1 cells were seeded at an initial concentration of 10^5 ^cells/mL in RPMI 1640 Medium supplemented with 2 mg/mL sodium bicarbonate (Atlanta Biologicals, GA, USA), 4.5 mg/mL glucose, 2 mg/mL HEPES, 1% Antibiotic-antimycotic, 10% serum fetal bovine. Cells were cultured at 37°C in an incubator with controlled humidified atmosphere containing 5% CO_2_. We detected apoptotic cells by 4,6-diamidino-2-phenylindole (DAPI; Sigma, St. Louis, MO, USA) staining. After washing with PBS and fixing with 4% paraformaldehyde for 15 min, the cells were stained with 2 mg/mL DAPI for 20 min. The stained cells were examined under a fluorescent microscope and cells were considered to undergo apoptosis based on the appearance of nuclear fragmentation.

### Detection of TNF-*α *activity by colorimetric sandwich ELISA

The induction agent, lipopolysaccharide (LPS) or F3, was dissolved in RPMI 1640 Medium at a stock concentration of 4 mg/mL and 2 mg/mL, respectively; then stored at -20°C. THP-1 cells with 10^5 ^cells/mL concentrations were seeded in 96-well microplates and incubated overnight. Then the cells (1.25 × 10^4^) were treated with F3 at the dosages indicated as 1 *μ*g/mL, 10 *μ*g/mL, 50 *μ*g/mL, 100 *μ*g/mL, 200 *μ*g/mL, and with LPS at the dose of 1 *μ*g/mL, respectively. The same volume of medium was applied as control. After 24 hours, the supernatants were collected at indicated time points by centrifugation at 1200 g for 5 min. In vitro TNF-*α *activity was determined using Human TNF-*α *Immunoassay Kit (Quantikine^®^, RD systems) based on the manual provided. The procedure is briefly described as follows. Assay Diluent RD1F solution (50 *μ*L) and 200 *μ*L of sample supernatants, standard and control, were gently mixed and loaded into individual wells. After 2 hours of incubation at room temperature, each well was aspirated, washed three times with 400 *μ*L of Wash Buffer, loaded with 200 *μ*l of secondary antibody solution (TNF-*α *Conjugate solution) at room temperature for 1 hour. The same aspiration and wash procedures were performed; 200 *μ*L of substrate solution was then added to each well and incubated in darkness at room temperature for 20 min. The enzymatic reaction was finally terminated by the addition of 50 *μ*l of Stop Solution. The optical density was determined using a microplate ELISA reader (Molecular Devices Corporation, California, USA) set at 450 nm with the correction wavelength at 540 or 570 nm. The concentration of TNF-*α *was determined by plotting the sample reading against the standard curve. All the measurements were performed in triplicate.

### Microarray gene expression analysis

THP-1 cells with 10^7 ^cells/mL concentrations were seeded in 100 mm dish and incubated overnight. After that, cells were treated with F3 and LPS at a final concentration of 30 *μ*g/mL and 1 *μ*g/mL, respectively. After incubated for 6 and 24 hours, the cell pellets were collected by centrifugation at 250 g for 5 min, correspondingly. Controlled samples of un-induced cells were treated in the same way with the same amount of medium. Total cellular RNA was extracted from a minimum of 5 × 10^6 ^cells with the use of TRIzol reagent (Invitrogen Life Technologies, Carlsbad, CA, USA); RNA was additionally purified with phenol-chloroform-isoamylalcohol (25:24:1). Purity was confirmed by spectrophotometry (A_260_/A_280 _ratio) and capillary electrophoresis (Agilent 2100 Bioanalyzer, Agilent Inc, Foster City, CA). RNA processing and hybridization onto Affymetrix (Santa Clara, CA) HG-U133A GeneChip oligonucleotide microarray were performed according to the manufacturer's protocol. Initial data analysis was performed using Affymetrix Microarray Suite v5.0 software, setting the scaling of all probe sets to a constant value of 500 for each GeneChip. Additional data analysis was performed using GeneSpring v5.1 (Silicon Genetics Inc., Redwood City, California). Principle component analysis on gene expression profiles of control (0 and 6 hours) and F3-treated (6 and 24 hours) samples were performed using Cluster 3.0 [62]. Genes with 2-fold change in differential expression between THP-1 control (combining 0 and 6 hours in total of four arrays) and F3- or LPS-treated THP-1 cells were selected for further analysis. Based on fold-change expression, genes that were differentially expressed between the two repeated microarray experiments were subjected to a scatter-plot analysis.

### Functional classification of differentially expressed genes

The differentially expressed genes were classified into groups based on their annotated functions using the BGSSJ software tool [63]. BGSSJ is an XML-based Java application that organizes lists of genes or proteins according to Gene Ontology database for biological interpretation [64], and organizes information based on molecular functions, biological processes, and cellular components for a number of different organisms. For our transcriptomic data, the UniGene IDs of the differentially expressed genes were used to input into BGSSJ with a database option of "NCBI + GO".

### Biological pathway annotations

The differentially expressed genes were annotated to specific biological pathways. For each UniGene ID, we retrieved its biological pathways from either BioCarta [44] or KEGG [45] through the existing NCI CGAP gene information database [46]. Biological pathways were mapped and sorted on the order of matching significance using the ArrayXPath web tool [47].

### Statistical analysis for identification of significantly disturbed pathways

The disturbed pathways with statistical significance were identified based on Fisher's exact test and false discovery rate. The statistical analysis was performed using ArrayXPath.

### Primer design

Primers were designed based on the published sequences in NCBI. We used Beacon Designer [65] to design the primers. Primers were designed to meet the following requirements: 75–150 bp in length, 50–60% in CG content, less than 5 degrees Tm difference between forward and reverse primers, limited GC repeats, amplicons between 80–120 bp, and limited dimer and hairpin formation. The designed primers are shown in Table 3.

**Table 3:** Primer sequences in Q-PCR experiments

Gene Name	Primer Sequence	PCR products
**Sense Primer**		
BCL2-S	CGATGTGATGCCTCTGCGAAG	95 bp
BCL2-A	GCCATGCTGATGTCTCTGGAATC	
BIRC2-S	GTTCCAGTTCAGCCTGAGCAG	102 bp
BIRC2-A	CCAACACCTCAAGCCACCATC	
CASP10-S	CACAGTCCACCCACCCTCTC	132 bp
CASP10-A	CTTCCTATGTGAGCACCTTCCTTAC	
FADD-S	ACAGCATCGAGGACAGATACCC	96 bp
FADD-A	CCACTGTTGCGTTCTCCTTCTC	
TNFRSF10B-S	GCACCACGACCAGAAACACAG	124 bp
TNFRSF10B-A	CAATCACCGACCTTGACCATCC	
TNFRSF12-S	TCAGCCAATGTGTCAGCAGTTC	150 bp
TNFRSF12-A	CGCAGCCATCGCCATGTTC	
TNFSF10-S	GCTGAAGCAGATGCAGGACAAG	136 bp
TNFSF10-A	CTGACGGAGTTGCCACTTGAC	
TNFSF12-S	TCAACAGCTCCAGCCCTCTG	146 bp
TNFSF12-A	CACACCATCCACCAGCAAGTC	
TRADD-S	TTTGCTGGCGGACGAGGAG	119 bp
TRADD-A	CCGAGCCGCACTTCAGATTTC	
CHUK-S	GCCATCCACTATGCTGAGGTTG	140 bp
CHUK-A	GCACGCTGTTCCAGAGATTCC	
NFKB1-S	CTCAAAGCAGCAGGAGCAGATC	71 bp
NFKB1-A	TCCCAAGAGTCATCCAGGTCATAG	
NFKBIA-S	AGTGATCCGCCAGGTGAAGG	130 bp
NFKBIA-A	ACAGCCAGCTCCCAGAAGTG	
RIPK1-S	AAGTGGGTGATGAGGGAAGGC	80 bp
RIPK1-A	TCGATCCTGGAACACTGGTGG	
TRAF2-S	AACATTGTCTGCGTCCTGAACC	159 bp
TRAF2-A	AGCCATCGCCAGGTCCTTG	
Casp3-S	ATGGACCACGCAGGAAGGG	68 bp
Casp3-A	GGCAGCATCATCCACACATACC	
Casp6-S	GCCACGCAGATGCCGATTG	139 bp
Casp6-A	CCAACCAGGCTGTGACACTTG	
Casp7-S	GTCTCACCTATCCTGCCCTCAC	114 bp
Casp7-A	TTCTTCTCCTGCCTCACTGTCC	
Casp8-S	GAAAAGCAAACCTCGGGGATAC	113 bp
Casp8-A	CCAAGTGTGTTCCATTCCTGTC	
GAPDH-S	ACACCCACTCCTCCACCTTTG	98 bp
GAPDH-A	GCTGTAGCCAAATTCGTTGTCATAC	

"S" means sense strand; "A" means anti-sense strand.

### Quantitative real-time PCR

Gene expression of the genes in death receptor pathway was determined by RT-PCR. Total RNA were isolated from cell lines using TRIzol reagent according to the manufacturer's protocol. First-strand cDNA synthesis was performed on 5 *μ*g of total RNA in a volume of 20 *μ*l with 1 *μ*l ThermoScript™ Reverse Transcriptase (Invitrogen) and 1 *μ*l oligo(dT). Extracted first-strand cDNAs were analyzed using a BioRad iCycler iQ Real-Time Detection System with SYBR Green dye (Molecular Probes, Eugene, OR). SYBR Green yields a strong fluorescent signal on binding double-stranded DNA enabling the quantification of gene expression by measurement of the intensity of the fluorescent light. SYBR Green will bind to any PCR product amplified by primers and is therefore, non-specific and less accurate than designing specialized probes. Targeted products were between 100–200 bp. For each experimental setup, a standard curve was prepared consisting of 5 dilutions of PCR products. Software produced for the BioRad iCycler will aid in evaluating collected data. mRNA expression of these genes were normalized to RNA content for each sample by using GADPH gene products as internal controls. The relative expression was calculated as the ratio of expression from each F3-treated THP-1 cells compared with the control THP-1 cells.

### Significance analysis of Q-PCR experiments

Significance analysis of Q-PCR experiments was performed by the EDGE software tool [54,66]. EDGE is based on Optimal Discovery Procedure, a new method that finds the optimal rule for calling differentially expressed genes, and Time Course Methodology.

### Western blotting

F3-treated and untreated THP-1 cells were washed with PBS twice. Cell pellets (1 × 10^7 ^cells) were solubilized in lysis buffer containing 7 M urea, 4% CHAPS, 2M thiourea, and 0.002% bromophenol blue. Lysates were centrifuged at 13,200 × g for 30 mins. Proteins were loaded into 10% SDS-PAGE and transferred onto polyvinylidene difluoride membranes (Millipore, Bedford, MA) at 150 V for 1.5 hours. After blocking in 5% nonfat milk in PBST containing 0.05% Tween 20 (Sigma) at room temperature overnight with gentle rocking, membranes were probed with antibodies. Primary antibodies involved in this study include CASP3 (IMGENEX, San Diego, CA) and CASP7 (Upstate, Lake Placid, NY) diluted in 1:200 and 1:300, respectively. Membranes were incubated with corresponding primary antibody and then incubated with secondary antibodies (biotinylated anti-mouse IgG-HRP, 1:2000 dilution, Abcam, Cambridge, UK). After incubation with secondary antibodies, immunoblots were visualized with the ECL detection kit (Amersham Biosciences) and exposed to X-ray film. *β*-actin was used as an internal loading control.

### Network construction

Based on the time-course Q-PCR data, we reconstructed a plausible gene regulatory network in F3-treated THP-1 cells using the BSIP web tool [55]. We developed BSIP to solve the reverse engineering problems for gene regulatory network or other biological networks. BSIP is a PHP-based web server for identification of biological networks using experimental time-series data obtained from microarray, Q-PCR, proteomics, or metabolomics measurements. Based on the S-system modeling formalism, X˙i=αi∏jXjgij−βi∏jXjhij
 MathType@MTEF@5@5@+=feaafiart1ev1aaatCvAUfKttLearuWrP9MDH5MBPbIqV92AaeXatLxBI9gBaebbnrfifHhDYfgasaacPC6xNi=xH8viVGI8Gi=hEeeu0xXdbba9frFj0xb9qqpG0dXdb9aspeI8k8fiI+fsY=rqGqVepae9pg0db9vqaiVgFr0xfr=xfr=xc9adbaqaaeGacaGaaiaabeqaaeqabiWaaaGcbaGafmiwaGLbaiaadaWgaaWcbaGaemyAaKgabeaakiabg2da9GGaciab=f7aHnaaBaaaleaacqWGPbqAaeqaaOWaaebuaeaacqWGybawdaqhaaWcbaGaemOAaOgabaGaem4zaC2aaSbaaWqaaiabdMgaPjabdQgaQbqabaaaaOGaeyOeI0caleaacqWGQbGAaeqaniabg+GivdGccqWFYoGydaWgaaWcbaGaemyAaKgabeaakmaarafabaGaemiwaG1aa0baaSqaaiabdQgaQbqaaiabdIgaOnaaBaaameaacqWGPbqAcqWGQbGAaeqaaaaaaSqaaiabdQgaQbqab0Gaey4dIunaaaa@4BEF@, where *X*_*i *_represents the gene expression level and *α*_*j*_, *β*_*j*_, *g*_*ij*_, and *h*_*ij *_represent the regulatory parameters, an evolutionary optimization method with data collocation [56] was used to estimate the modeling parameters with the measured time-series data of gene expression and determine the regulatory network structure.

## Authors' contributions

KCC carried out cell culture, detection of TNF-*α *activity, Q-PCR and microarray experiments. KCC, HCH, JHC, JWH, HCC and HFJ undertook microarray data analysis. HCH and CHO constructed the gene network and biological pathways. JWH, carried out the western blotting and DAPI experiments. WBY, STC and CHW purified and provided F3 polysaccharides. HCH and HFJ designed the study. HFJ, drafted the manuscript, conceived and directed the project. All authors read and approved the final manuscript.
